# Negative effects of nitrogen fertilization on herbivore fitness are exaggerated at warmer temperatures and in high-altitude populations

**DOI:** 10.1007/s00442-025-05690-8

**Published:** 2025-03-11

**Authors:** Ange Raharivololoniaina, Roland Busch, Franziska Deppe, Anna Hitzler, Eva Plath, Tamara Rischen, Mine Yilmazer, Klaus Fischer

**Affiliations:** Department of Biology, Institute for Integrated Natural Sciences, University of Koblenz, Universitätsstraße 1, 56070 Koblenz, Germany

**Keywords:** Biodiversity conservation, Butterfly, Eutrophication, Global change, Plant–herbivore interaction

## Abstract

Biodiversity is currently under strong pressure due to anthropogenic global change. Different drivers of global change may exert direct and indirect effects on biodiversity, and may furthermore interact with one another, but our respective knowledge is still very limited. We investigated indirect and interactive effects of two important drivers of global change, eutrophication and climate change, in replicated low- and high-altitude populations of an insect herbivore, the butterfly *Lycaena tityrus*, in a laboratory setting. We found local adaptation in developmental traits, with low-altitude populations being adapted to warmer temperatures and longer seasons. *Lycaena tityrus* responded negatively to agriculturally relevant levels of fertilization of its host plant, showing reduced body mass and prolonged development time. Negative effects were particularly pronounced at warmer temperatures and in high-altitude populations. Our study adds to the increasing knowledge that different drivers of global change may interact and thereby increase the overall level of threat to biodiversity. We suggest that populations inhabiting naturally nutrient-poor environments might be even more vulnerable to agricultural intensification than others, potentially applicable to many species. These findings may have important implications for protecting numerous vulnerable species in the face of rapid environmental change.

**Supplementary Information:**

The online version contains supplementary material available at 10.1007/s00442-025-05690-8.

## Introduction

In the current era, biodiversity is strongly threatened by global environmental change, which involves a variety of human-induced drivers (Pereira et al. 2012; Ceballos et al. 2015; Johnson et al. 2017; Wagner 2020). These drivers may directly or indirectly affect biodiversity, and may additionally interact with one another (Oliver and Morecroft 2014; Jackson et al. 2016; Gunderson et al. 2017; Hamann et al. 2021). While the direct effects of individual drivers have been quite intensively studied, our knowledge on indirect effects and interactions among drivers is still limited (Oliver and Morecroft 2014; Sage 2020; Wagner 2020; Kuczyk et al. 2021a).

Important drivers of biodiversity loss are agricultural intensification and anthropogenic climate change (Pereira et al. 2012; Ceballos et al. 2015; WallisDeVries and Van Swaay 2017). With regard to agricultural intensification, adverse effects of eutrophication on species richness have been widely documented for plants (Bobbink et al. 2010; Stevens et al. 2010), but also for invertebrates (Van Dyck et al. 2009; Carvalheiro et al. 2019; Nessel et al. 2021). Negative effects of excess nitrogen on animals might be conveyed by microclimatic cooling, reduced host-plant quality, and the loss of host plants or nectar sources (Fischer and Fiedler 2000; WallisDeVries and Van Swaay 2006; Lebeau et al. 2016; Nijssen et al. 2017). However, our knowledge on the effects of nitrogen fertilization on animals is still limited (Nijssen et al. 2017). With regard to anthropogenic climate change, its effects on biodiversity have been widely demonstrated and discussed (Weiskopf et al. 2020; Pereira et al. 2024). Climate change may induce shifts in phenology or geographic ranges, and may also affect morphology, life history and behavior (Scheffers et al. 2016; Weiskopf et al. 2020). Both, eutrophication and climate change, likely play a significant role in biodiversity loss, specifically in the decline of insect diversity and abundance (Nessel et al. 2021; Raven and Wagner 2021).

Herbivores comprise particularly interesting organisms to study the consequences of global change, as they are sensitive to direct as well as indirect effects, the latter being mediated by alterations in host-plant quality or availability (Harvey et al. 2020; Kuczyk et al. 2021a). For instance, increased temperatures during plant growth may diminish host-plant quality for insect herbivores (Bauerfeind and Fischer 2013; Kuczyk et al. 2021a, b), which may result from increased C:N ratios, concentrations of secondary metabolites, and leaf toughness (Meihls et al. 2012; Block et al. 2019). Similarly, nitrogen fertilization, as abundantly applied in modern agriculture, affects host-plant chemistry (Kuczyk et al. 2021a, b). This may in turn diminish herbivore performance, e.g. by reducing survival rates, growth rates and body mass (Fischer and Fiedler 2000; Kurze et al. 2018; Raharivololoniaina et al. 2023).

In species with a wide geographical distribution, populations may respond differently to environmental stressors (Kuczyk et al. 2021b). Such within-species variation in the vulnerability to global change has been largely neglected thus far, although geographical variation in trait values is common (Kawecki and Ebert 2004; Blanquart et al. 2013). For instance, in the widespread butterfly *Pieris napi*, fast-growing Italian populations are more sensitive to poor food quality than slower growing German ones (Kuczyk et al. 2021b). Such studies on local adaptation provide important insights into the power of natural selection relative to gene flow and other evolutionary forces (Kawecki and Ebert 2004), but add an additional level of complexity.

Here, we study indirect and interactive effects of two important global change drivers, namely eutrophication and a vital component of climate change, viz. increasing temperature. Specifically, we investigate effects of host-plant nitrogen fertilization and herbivore developmental temperature, on high- and low-altitude populations of the butterfly *Lycaena tityrus* (Poda 1761). This species has been repeatedly used as a model to study phenotypic plasticity, local and thermal adaptation as well as dispersal (e.g., Karl et al. 2008, 2009a, b; Fischer and Karl 2010; Fischer et al. 2011; Reim et al. 2018a, b). These studies revealed, amongst others, that high- compared to low-altitude populations showed longer development times, increased cold- but decreased heat-stress resistance, reduced plasticity in heat resistance, temperature-induced plasticity in developmental traits, and a pronounced sexual dimorphism. In terms of host-plant quality, effects of nitrogen fertilization of host plants (Fischer and Fiedler 2000; Kurze et al. 2018), and nitrogen fertilization in combination with drought stress (Raharivololoniaina et al. 2023) or high plant growing temperature have been investigated (Raharivololoniaina et al. 2021). Results showed detrimental effects of high levels of host-plant fertilization and of drought stress on the species’ performance, but positive effects of limited nitrogen fertilization and of higher plant rearing temperatures. However, earlier studies have not investigated effects of host-plant nitrogen fertilization (1) across altitudes or (2) at different insect developmental temperatures, which are the aims of the current study.

We predict that (1) cold-adapted high-altitude populations of *L. tityrus*, which are not (yet) exposed to high levels of eutrophication, are more sensitive to host plant fertilization than low-altitude populations, and that (2) detrimental effects of fertilization are more pronounced at higher ambient temperatures, indicating an interactive effect between two important drivers of biodiversity loss, eutrophication and climate change.

## Materials and methods

### Study organism

*Lycaena tityrus* (Poda 1761) is a widespread, temperate-zone butterfly ranging from Spain throughout large parts of central and southern Europe to western Asia (Ebert and Rennwald 1991; Bräu et al. 2013). In most parts of its range, it has 2–3 generations per year (Ebert and Rennwald 1991; Tolman and Lewington 2008). Two subspecies are recognized in central Europe, the widespread nominate form *L. tityrus tityrus* (Poda 1761) and the alpine subspecies *L. tityrus subalpinus* (Speyer 1851). The latter is confined to the higher altitudes of the European Alps and some other mountain ranges, where it has only one generation per year (Tolman and Lewington 2008). The altitudinal distribution of *L. tityrus subalpinus* ranges from 1200 to 2500 m a.s.l. (Tolman and Lewington 2008).

*Lycaena tityrus* inhabits humid to moderately dry locations such as low-intensity grassland, alpine meadows, sandy heathland, bogs, and forest edges (Ebert and Rennwald 1991; Bräu et al. 2013). It uses different species of sorrel (*Rumex* spec., Polygonaceae) as larval host plant (Ebert and Rennwald 1991; Tolman and Lewington 2008). The primary hostplant is *Rumex acetosa* L., which is a perennial herb containing a variety of primarily non-nitrogenous secondary compounds, especially oxalic acids (Korpelainen and Pietiläinen 2020). Adults are rather opportunistic nectar feeders, using a wide array of plants (Ebert and Rennwald 1991). *Lycaena tityrus* has suffered substantial population losses due to grassland intensification, meadow conversion, and afforestation in recent decades (Bräu et al. 2013). It is projected to shift its distributional range northward and upward due to anthropogenic climate change (Settele et al. 2008).

### Population sampling

For this study, we collected in 2022 fecund females from four replicated low-altitude populations in northeastern and western Germany (*L. tityrus tityrus*; Greifswald, coordinates: 54.05°N, 13.44°E; Altwarp: 53.74°N, 14.26°E; Pottum: 50.59°N, 8.00°E; Westerburg: 50.54°N, 7.97°E; altitudinal range 0–420 m a.s.l.), and from four replicated high-altitude populations in Austria (*L. tityrus subalpinus*; Kühtai: 47.23°N, 10.97°E; Obernberg: 46.98°N, 11.42°E; Obergurgl: 46.86°N, 11.02°E; Venn: 47.01°N, 11.54°E; altitudinal range 1560–2030 m a.s.l.). All high-altitude populations were sampled on unfertilized alpine meadows in the central Alps, i.e., above crystalline primary rock (gneiss, slate) resulting in acidic, nutrient-poor soils. The low-altitude populations, in contrast, originate from slightly fertilized meadows from areas that are also naturally richer in nutrients (basalt mountains or marshland). Note that sampling locations not only differed in altitude but also in latitude. However, the impact of variation in altitude is expected to surpass the potential impact of latitude by far (Karl et al. 2008, 2009a). Per population, we sampled between 6 and 20 females, in total 34 from low- and 48 from high-altitude populations. Females were afterwards transferred to and randomly distributed among two climate cabinets (26 °C, 70% humidity, L19:D5 photoperiod; Panasonic MIR-554) at Koblenz University for egg laying. Therefore, females were kept individually in translucent plastic pots (1 L) covered with gauze. They were provided sucrose solution and fresh flowers for adult feeding, and leaves of *Rumex acetosa* as oviposition substrate. Deposited eggs were collected daily and transferred, separated by female, to small glass vials kept in one climate cabinet at 22 °C (70% humidity, L19:D5 throughout) until hatching (Panasonic MIR-554). Hatchlings were fed on field-collected *R. acetosa* leaves under the same conditions for 4 days, after which they were allocated to treatments.

### Experimental design

On day 5 after hatching, larvae were individually transferred to transparent plastic boxes (250 ml) lined with moist tissue and containing a fresh cutting of *R. acetosa*. Larvae were randomly allocated to one out of four treatments, using a split-brood design. We employed a full-factorial design with two rearing temperatures and two host plant treatments. Rearing temperatures had a mean of 19.2 °C (light phase 20 °C, dark phase 16 °C; L19:D5 photoperiod) or 25.2 °C (light phase 26 °C, dark phase 22 °C), and we used one climate cabinet per temperature (Panasonic MIR-554). To rule out effects of individual cabinets, temperature regimes and according larvae were shifted among both cabinets every four days. Host plant treatments involved low versus high levels of nitrogen fertilization, the latter mimicking levels typically used in modern agriculture (Raharivololoniaina et al. 2021, 2023). Host plants, 200 per treatment, were grown individually in standard pots (9 × 9 × 10 cm, 0.5 L) in a greenhouse from commercially available seeds. Plants were provided with a common nitrogenous fertilizer (NH_4_NO_3_), and received a total amount of 1.225 ml (low N) or 3.675 ml (high N) of NH_4_NO_3_. The total amount of NH_4_NO_3_ was equally divided into five applications of 0.245 ml (low N) or 0.735 ml (high N) in 100 ml water each, corresponding to one application per week during the larval feeding phase. These nitrogen treatments have been shown to be highly effective in changing plant chemistry; specifically, higher nitrogen levels resulted in higher nitrogen and water contents but a reduced C:N ratio (Raharivololoniaina et al. 2021, 2023). All boxes were cleaned daily and supplied with a fresh leaf cutting and moist tissue. Leaf cuttings were taken from at least 20 plants per day and randomly allocated to the larvae within treatments. Boxes were shifted around daily to minimize potential temperature and light differences within the climate cabinets. All offspring (*n* = 579) were reared at the treatment they were allocated to until adult eclosion. Afterwards, the sex of each individual was determined and the animals frozen at -18 °C for later analyses.

### Data collection and laboratory analyses

For each individual, we scored different developmental and morphological traits as well as mortality. Larval and pupal development time were measured in days. Pupal mass was weighed on the day following pupation to the nearest 0.1 mg (KERN ABJ-NM/ABS-N). Larval growth rate was calculated as mean weight gain per day (LN (pupal mass)/larval time). Frozen butterflies were first weighed to obtain total adult mass. Then, wings, head and legs were removed, and the abdomen was separated from the thorax. Abdomen and thorax were weighed separately, and thorax-abdomen ratio was calculated as a measure of the relative investment into flight versus reproduction. The length and area of the right forewings were measured using digital images and the software ImageJ. Wing loading was calculated as adult body mass divided by forewing area, and wing aspect ratio as 4 × forewing length^2^ divided by forewing area (Berwaerts et al. 2002). Finally, abdomen fat content was quantified, but due to the high work load only in a subset of individuals, following Fischer et al. (2003). In short, abdomens were dried at 60 °C for 24 h and then weighed. Thereafter, fat was extracted from the dried abdomens for 48 h on a laboratory shaker using 1.5 mL of dichloromethane (CH_2_CL_2_) for each abdomen. The extraction was repeated once, after which the abdomens were once again dried at 60 °C for 24 h and afterwards weighed. Abdomen fat content was then calculated by subtracting the fat-free abdomen dry mass from the initial abdomen dry mass and is given in percent.

### Statistical analyses

We used general linear mixed models (GLMMs) to analyze all traits obtained (except survival, see below), with origin (low versus high altitude), sex, developmental temperature, and host-plant nitrogen treatment as fixed factors, and replicate population (nested within origin) and family (the offspring of a given female; nested within origin and population) as random factors. Minimum adequate models were constructed by sequentially removing non-significant interaction terms, in case no higher order interactions were significant. Larval time, pupal time, and larval growth rate were LN-transformed prior to analyses to meet GLMM requirements. Survival data were analyzed using a binary logistic regression with the same factors and model structure as above. All tests were computed using Statistica 12.0 (Statsoft, Tulsa, USA) or R 4.4.2 (R Core Team 2024) using the ‘lme4’ package (Bates et al. 2015). Throughout the text, we present means ± standard error.

## Results

Low- and high-altitude populations differed significantly in larval time, pupal time, larval growth rate, wing length, wing area, wing loading, wing aspect ratio, and abdomen fat content (Table 1). High- compared with low-altitude populations showed longer larval times (33.4 ± 0.4 vs. 28.4 ± 0.4 d), shorter pupal times (10.5 ± 0.1 vs. 11.5 ± 0.1 d), lower larval growth rates (4.30 ± 0.10 vs. 5.04 ± 0.08 mg/d), shorter forewings (14.44 ± 0.06 vs. 15.26 ± 0.05 mm), smaller forewing areas (88.0 ± 0.8 vs. 96.5 ± 0.6 mm^2^), higher wing loadings (0.369 ± 0.006 vs. 0.350 ± 0.005 mg/mm^2^), and lower wing aspect ratios (9.59 ± 0.06 vs. 9.72 ± 0.04) and abdomen fat contents (9.6 ± 0.9 vs. 17.3 ± 0.7%). In addition, thorax masses tended to be lower in high- versus low-altitude populations (14.7 ± 0.3 vs. 15.5 ± 0.2 mg).

**Table 1:** Results of general linear mixed models for the effects of origin (low vs. high altitude), sex, rearing temperature, host-plant nitrogen fertilization (low vs. high; all fixed), replicate population (random, nested within origin), and family (random, nested within population and origin) on various traits of the butterfly *Lycaena tityrus*

Source	Effect	DFn	DFd	MS	*F*	*p*
Larval time						
Origin	Fixed	1	8	0.59	5.63	**0.0470**
Sex	Fixed	1	534	1.28	30.72	**< 0.0001**
Temperature	Fixed	1	534	50.03	1205.15	**< 0.0001**
Nitrogen	Fixed	1	534	0.30	7.15	**0.0077**
Origin*Temp	Fixed	1	534	1.04	25.00	**< 0.0001**
Origin*Nitro	Fixed	1	534	0.34	8.18	**0.0044**
Population (Origin)	Random	6	78	0.13	2.65	**0.0217**
Family (Origin*Pop)	Random	31	534	0.05	1.28	0.1472
Error		534		0.04		
Pupal time						
Origin	Fixed	1	7	0.39	24.68	**0.0015**
Sex	Fixed	1	525	0.39	45.01	**< 0.0001**
Temperature	Fixed	1	525	20.63	2402.60	**< 0.0001**
Nitrogen	Fixed	1	525	< 0.01	0.06	0.8133
Population (Origin)	Random	6	64	0.02	1.33	0.2563
Family (Origin*Pop)	Random	31	525	0.02	1.82	**0.0049**
Error		525		0.01		
Pupal mass						
Origin	Fixed	1	8	1175.78	1.88	0.2066
Sex	Fixed	1	529	7075.64	24.21	**< 0.0001**
Temperature	Fixed	1	529	694.77	2.38	0.1237
Nitrogen	Fixed	1	529	367.53	1.26	0.2626
Origin*Sex	Fixed	1	529	21.96	0.08	0.7841
Origin*Temp	Fixed	1	529	1051.88	3.60	0.0584
Sex*Temp	Fixed	1	529	1.29	< 0.01	0.9470
Origin*Nitro	Fixed	1	529	337.25	1.15	0.2832
Sex*Nitro	Fixed	1	529	510.61	1.75	0.1868
Temp*Nitro	Fixed	1	529	819.63	2.80	0.0946
Origin*Temp*Nitro	Fixed	1	529	1297.52	4.44	**0.0356**
Population (Origin)	Random	6	54	732.40	1.40	0.2303
Family (Origin*Pop)	Random	31	529	698.63	2.39	**0.0001**
Error		529		292.28		
Larval growth rate						
Origin	Fixed	1	8	1.19	11.60	**0.0094**
Sex	Fixed	1	531	0.24	4.87	**0.0277**
Temperature	Fixed	1	531	49.76	1015.84	**< 0.0001**
Nitrogen	Fixed	1	531	0.18	3.67	0.0560
Origin*Temp	Fixed	1	531	0.59	12.14	**0.0005**
Origin*Nitro	Fixed	1	531	0.29	5.85	**0.0159**
Population (Origin)	Random	6	81	0.12	2.22	**0.0494**
Family (Origin*Pop)	Random	31	531	0.06	1.20	0.2111
Error		531		0.05		
Adult mass						
Origin	Fixed	1	15	19.93	0.43	0.5237
Sex	Fixed	1	532	10,338.96	187.37	**< 0.0001**
Temperature	Fixed	1	532	6496.44	117.73	**< 0.0001**
Nitrogen	Fixed	1	532	297.57	5.39	**0.0206**
Origin*Temp	Fixed	1	532	5645.05	102.30	**< 0.0001**
Population (Origin)	Random	6	50	39.72	0.34	0.9114
Family (Origin*Pop)	Random	31	532	165.61	3.00	**< 0.0001**
Error		532		55.18		
Thorax mass						
Origin	Fixed	1	15	48.44	4.54	0.0504
Sex	Fixed	1	533	83.11	6.27	**0.0125**
Temperature	Fixed	1	533	1079.83	81.53	**0.0000**
Nitrogen	Fixed	1	533	18.84	1.42	0.2336
Origin*Sex	Fixed	1	533	87.40	6.60	**0.0105**
Origin*Temp	Fixed	1	533	960.27	72.50	**< 0.0001**
Temp*Nitro	Fixed	1	533	54.66	4.13	**0.0427**
Population (Origin)	Random	6	54	9.20	0.40	0.8776
Family (Origin*Pop)	Random	31	533	30.74	2.32	**0.0001**
Error		533		13.25		
Abdomen mass						
Origin	Fixed	1	9	2.44	0.13	0.7318
Sex	Fixed	1	535	6287.01	601.96	**< 0.0001**
Temperature	Fixed	1	535	1336.71	127.99	**< 0.0001**
Nitrogen	Fixed	1	535	47.68	4.56	**0.0331**
Origin*Temp	Fixed	1	535	560.00	53.62	**< 0.0001**
Sex*Temp	Fixed	1	535	110.82	10.61	**0.0012**
Population (Origin)	Random	6	49	22.12	1.00	0.4343
Family (Origin*Pop)	Random	31	535	30.97	2.97	**< 0.0001**
Error		535		10.44		
Thorax-abdomen ratio						
Origin	Fixed	1	7	0.44	0.71	0.4290
Sex	Fixed	1	536	136.19	727.77	**< 0.0001**
Temperature	Fixed	1	536	2.39	12.77	**0.0004**
Nitrogen	Fixed	1	536	0.18	0.95	0.3296
Population (Origin)	Random	6	63	0.68	2.52	**0.0298**
Family (Origin*Pop)	Random	31	536	0.33	1.77	**0.0069**
Error		536		0.19		
Wing length						
Origin	Fixed	1	10	28.75	36.56	**0.0001**
Sex	Fixed	1	526	2.69	4.70	**0.0306**
Temperature	Fixed	1	526	8.16	14.26	**0.0002**
Nitrogen	Fixed	1	526	< 0.01	< 0.01	0.9667
Origin*Sex	Fixed	1	526	4.63	8.09	**0.0046**
Origin*Temp	Fixed	1	526	0.48	0.85	0.3580
Sex*Temp	Fixed	1	526	2.29	4.00	**0.0460**
Origin*Nitro	Fixed	1	526	1.05	1.84	0.1755
Sex*Nitro	Fixed	1	526	0.22	0.39	0.5344
Temp*Nitro	Fixed	1	526	3.58	6.25	**0.0127**
Origin*Temp*Nitro	Fixed	1	526	3.16	5.52	**0.0192**
Population (Origin)	Random	6	52	0.84	0.76	0.6010
Family (Origin*Pop)	Random	31	526	1.50	2.62	**< 0.0001**
Error		526		0.57		
Wing area						
Origin	Fixed	1	9	2724.44	21.46	**0.0011**
Sex	Fixed	1	529	10,610.74	125.16	**< 0.0001**
Temperature	Fixed	1	529	393.02	4.64	**0.0318**
Nitrogen	Fixed	1	529	261.25	3.08	0.0798
Temp*Nitro	Fixed	1	529	872.54	10.29	**0.0014**
Origin*Temp*Nitro	Fixed	3	529	246.84	2.91	**0.0340**
Population (Origin)	Random	6	55	138.86	0.95	0.4643
Family (Origin*Pop)	Random	31	529	191.55	2.26	**0.0002**
Error		529		84.78		
Wing loading						
Origin	Fixed	1	28	0.02	8.96	**0.0057**
Sex	Fixed	1	527	0.55	115.06	**< 0.0001**
Temperature	Fixed	1	527	0.74	155.66	**< 0.0001**
Nitrogen	Fixed	1	527	0.04	7.87	**0.0052**
Origin*Sex	Fixed	1	527	0.07	15.31	**0.0001**
Origin*Temp	Fixed	1	527	0.73	153.70	**< 0.0001**
Sex*Temp	Fixed	1	527	0.03	5.85	**0.0159**
Population (Origin)	Random	6	52	< 0.01	0.18	0.9805
Family (Origin*Pop)	Random	31	527	0.01	2.66	**< 0.0001**
Error		527		< 0.01		
Wing aspect ratio						
Origin	Fixed	1	13	2.33	10.70	**0.0063**
Sex	Fixed	1	532	48.57	99.97	**< 0.0001**
Temperature	Fixed	1	532	4.08	8.39	**0.0039**
Nitrogen	Fixed	1	532	2.81	5.79	**0.0165**
Sex*Temp	Fixed	1	532	2.19	4.50	**0.0344**
Population (Origin)	Random	6	71	0.17	0.28	0.9428
Family (Origin*Pop)	Random	31	532	0.69	1.43	0.0643
Error		532		0.49		
Fat content						
Origin	Fixed	1	7	1735.72	11.44	**0.0110**
Sex	Fixed	1	315	311.61	3.69	0.0556
Temperature	Fixed	1	315	119.53	1.42	0.2349
Nitrogen	Fixed	1	315	141.32	1.67	0.1966
Population (Origin)	Random	6	105	166.85	2.23	**0.0456**
Family (Origin*Pop)	Random	27	315	66.58	0.79	0.7662
Error		315		84.39		

Minimum adequate models were constructed by sequentially removing non-significant interaction terms, as far as no higher order interactions were significant. Significant *p*-values are given in bold

**Fig. 1:**
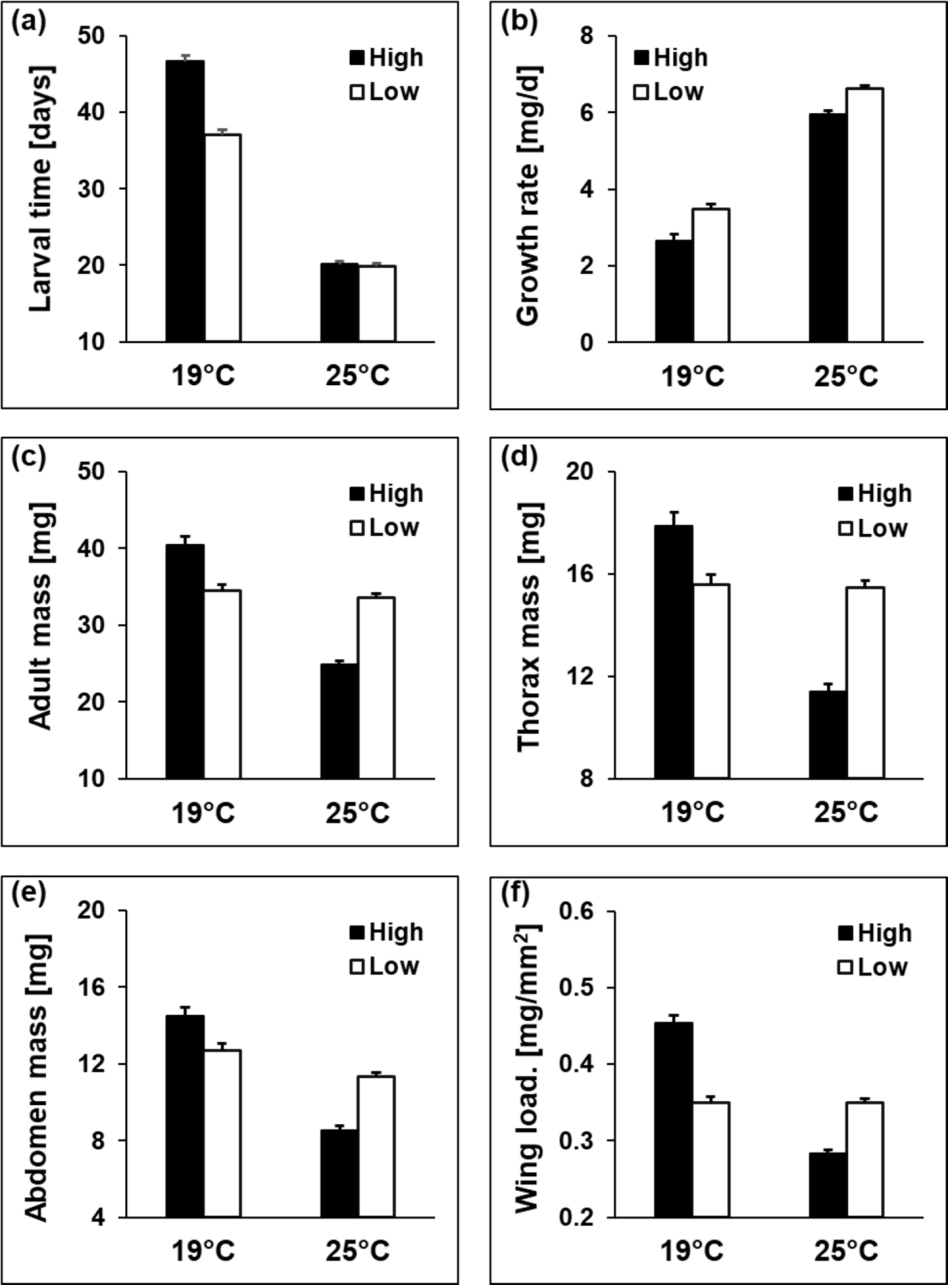
Larval time (**a**), larval growth rate (**b**), adult mass (**c**), thorax mass (**d**), abdomen mass (**e**), and wing loading (**f**) in relation to origin (high vs. low altitude) and rearing temperature (19 vs. 25 °C) in the butterfly *Lycaena tityrus*. Given are means ± SE

**Fig. 2:**
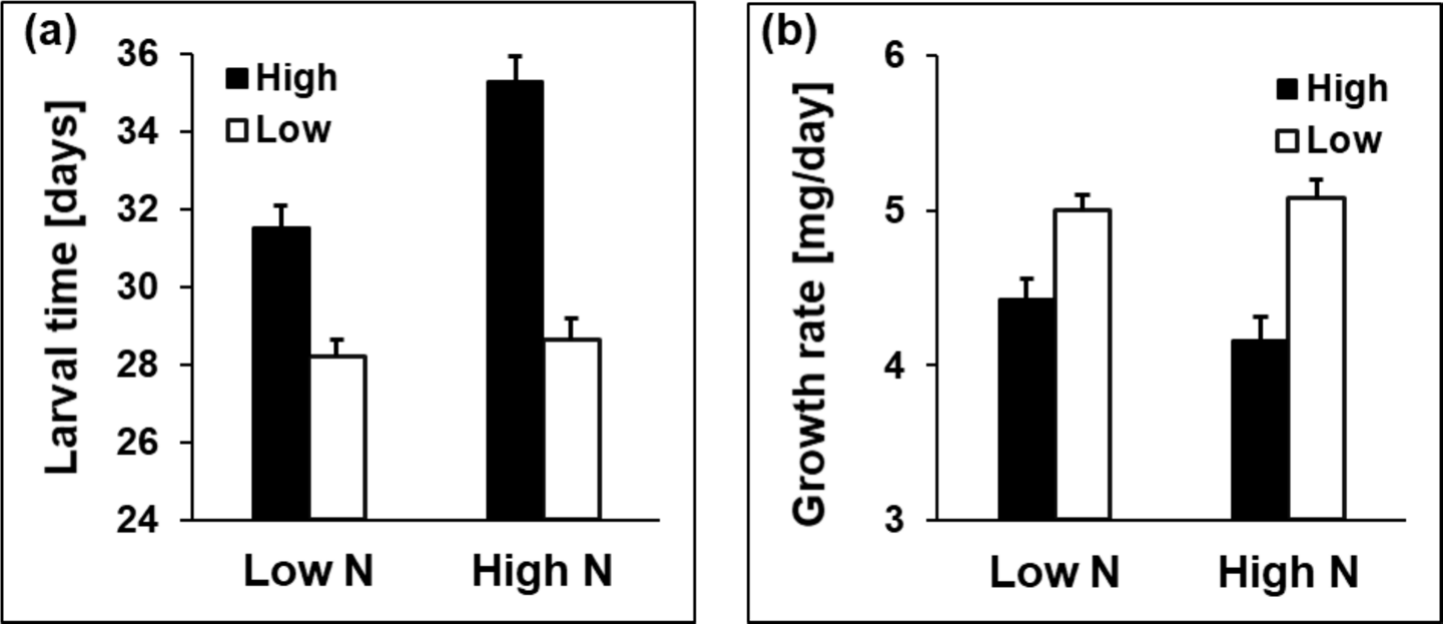
Larval time (**a**) and larval growth rate (**b**) in relation to origin (high vs. low altitude) and host-plant nitrogen fertilization (low N vs. high N) in the butterfly *Lycaena tityrus*. Given are means ± SE

The above general patterns were modulated by interactions with other factors (Table 1). For six traits, the interaction between origin and rearing temperature was significant. These interactions show for larval time and larval growth rate that differences among altitudes were restricted to or more pronounced at the lower temperature (Fig. 1). Adult, thorax and abdomen mass were higher in high-altitude populations at the lower rearing temperature, but lower at the higher rearing temperature. Accordingly, wing loading was higher in high- than in low-altitude populations at the lower temperature, but the other way around at the higher temperature (Fig. 1f). The significant origin by nitrogen interactions for larval time and larval growth rate show that the high-altitude populations responded much more strongly to the high nitrogen treatment, prolonging larval time and reducing larval growth rates (Fig. 2).

All measured traits differed significantly among sexes except from fat content, for which there was only a tendency towards a lower fat content in females (12.5 ± 0.8 vs. 14.4 ± 0.7%). Females compared with males had longer larval (32.2 ± 0.4 vs. 29.6 ± 0.4 d) and pupal times (11.2 ± 0.1 vs. 10.7 ± 0.1 d), higher pupal masses (126.5 ± 1.3 vs. 118.2 ± 1.2 mg), lower larval growth rates (4.57 ± 0.10 vs. 4.77 ± 0.10 mg/d), higher adult (38.2 ± 0.6 vs. 28.5 ± 0.5 mg), thorax (15.5 ± 0.3 vs. 14.7 ± 0.3 mg) and abdomen masses (15.6 ± 0.3 vs. 7.9 ± 0.2 mg), lower thorax-abdomen ratios (1.04 ± 0.03 vs. 2.00 ± 0.03), longer wings (15.0 ± 0.1 vs. 14.8 ± 0.1 mm), larger wing areas (96.6 ± 0.7 vs. 87.9 ± 0.7 mm^2^), higher wing loadings (0.395 ± 0.005 vs. 0.323 ± 0.005 mg/mm^2^), and lower wing aspect ratios (9.33 ± 0.05 vs. 9.99 ± 0.05). The interaction between origin and sex was significant for thorax mass, wing length, and wing loading, showing that sex differences were restricted to or more pronounced in high-altitude (thorax mass, wing loading) or low-altitude populations (wing length; Table S1b, supplementary material).

**Fig. 3:**
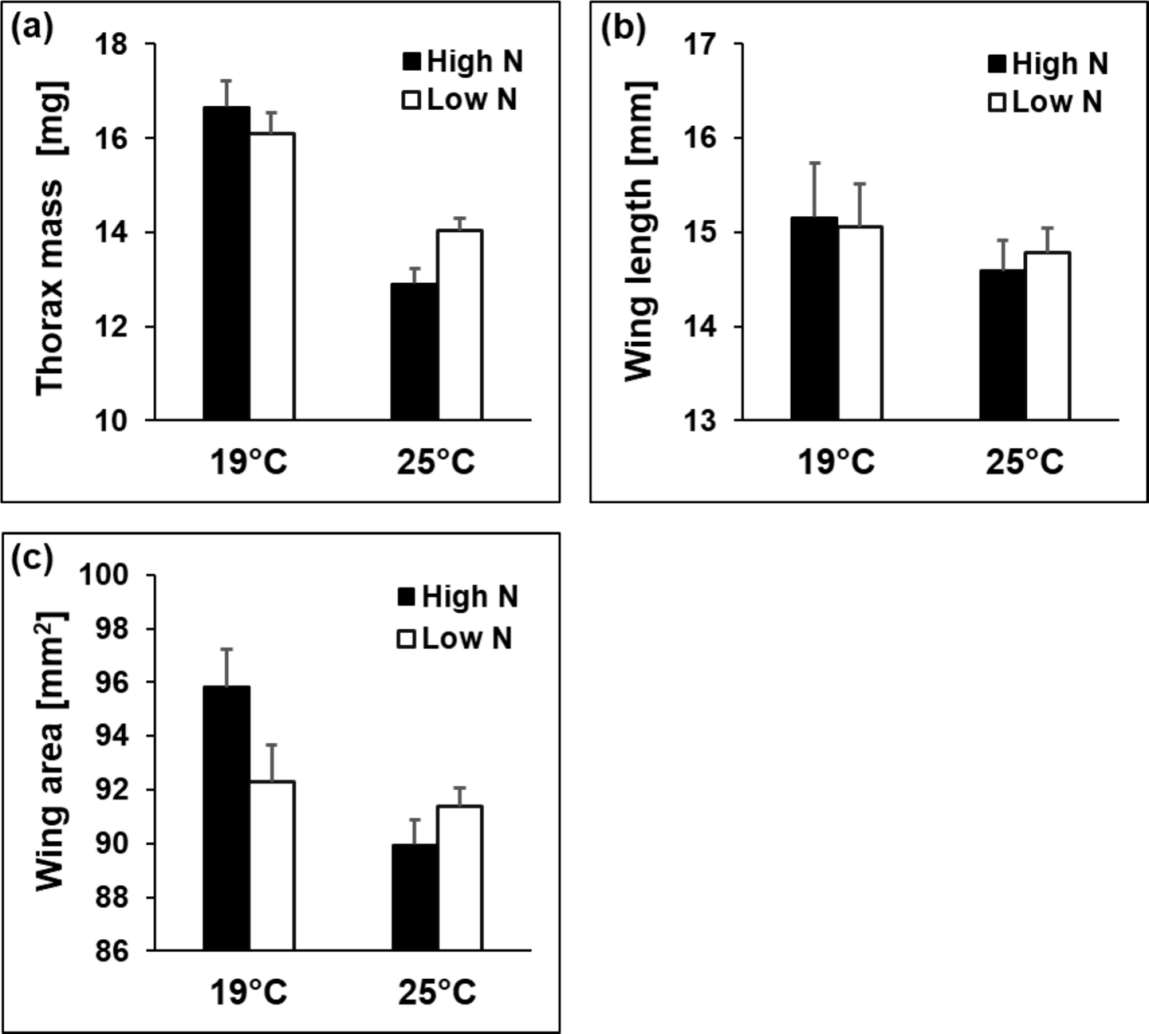
Thorax mass (**a**), wing length (**b**), and wing area (**c**) in relation to temperature (19 vs. 25 °C) and host-plant nitrogen treatment (low N vs. high N) in the butterfly *Lycaena tityrus*. Given are means ± SE

All traits except from pupal mass and abdomen fat content differed among rearing temperatures. At the lower rearing temperature, larval (41.9 ± 0.5 vs. 20.0 ± 0.3 d) and pupal times (13.4 ± 0.1 vs. 8.5 ± 0.1 d) were longer, larval growth rate (3.06 ± 0.11 vs. 6.28 ± 0.06 mg/d) was lower, adult (37.5 ± 0.7 vs. 29.2 ± 0.4 mg), thorax (16.7 ± 0.3 vs. 13.5 ± 0.2 mg) and abdomen masses (13.6 ± 0.3 vs. 9.9 ± 0.2 mg) were higher, thorax-abdomen ratio (1.42 ± 0.04 vs. 1.59 ± 0.02) was lower, wings (15.0 ± 0.1 vs. 14.7 ± 0.1 mm) were longer, wing area (93.3 ± 0.8 vs. 91.1 ± 0.5 mm^2^) was larger, and wing loading (0.402 ± 0.006 vs. 0.316 ± 0.004 mg/mm^2^) and wing aspect ratio (9.77 ± 0.06 vs. 9.54 ± 0.04) were higher. The reductions in abdomen mass and concomitantly wing loading at the higher temperature were more pronounced in females than in males. However, the reductions in wing length and wing aspect ratio at the higher temperature were more pronounced in males than in females (significant sex by temperature interactions; Table S1a, supplementary material). The reduction in thorax mass, wing length and area at the higher temperature were more pronounced in the high nitrogen treatment (significant temperature by nitrogen treatment interactions; Fig. 3). For the latter two traits, this effect was especially pronounced in high altitude populations (significant three-way interactions; Table S2, supplementary material).

Host-plant nitrogen treatment significantly affected larval time, adult mass, abdomen mass, wing loading, and wing aspect ratio. At the high as compared with the low nitrogen treatment larval time (32.0 ± 0.4 vs. 29.9 ± 0.4 d) was longer, adult (33.1 ± 0.6 vs. 33.6 ± 0.5 mg) and abdomen masses (11.6 ± 0.3 vs. 12.0 ± 0.2 mg) were lower, and wing loading (0.351 ± 0.006 vs. 0.368 ± 0.005 mg/mm^2^) and wing aspect ratio (9.54 ± 0.05 vs. 9.76 ± 0.05) were lower. Additionally, larval growth rate tended to be lower in the high nitrogen treatment (4.62 ± 0.10 vs. 4.72 ± 0.08 mg/day). The significant origin by temperature by nitrogen interaction indicates that nitrogen fertilization had generally little impact on pupal mass, except from the high-altitude populations at the lower rearing temperature, where high nitrogen resulted in heavier pupae (Table S2, supplementary material).

Significant variation between replicate populations were found in larval time, larval growth rate, thorax-abdomen ratio, and fat content, and between families in pupal time, pupal mass, adult mass, thorax mass, abdomen mass, thorax-abdomen ratio, wing length, wing area, and wing loading (Table 1). This suggests genetic variation in development time, body size, storage reserves, and the allocation to dispersal versus reproduction among populations and / or families.

Survival rates, finally, only differed significantly among rearing temperatures (19 °C: 55.1%, 25 °C: 85.2%; *z* = − 4.55, p < 0.0001), but not in relation to origin (low altitude: 84.0%, high altitude: 72.2%; *z* = − 1.37, *p* = 0.1715) or nitrogen treatment (low N: 84.2%, high N: 71.1%; *z* = − 1.06, *p* = 0.2884). All interactions were non-significant (all *p*-values > 0.14).

## Discussion

Our study revealed evidence for local genetic adaptation in high- and low-altitude populations of the butterfly *Lycaena tityrus*. Specifically, low-altitude populations showed an overall shorter development time associated with higher larval growth rates (cf. Karl et al. 2008), presumably as an adaptation to having 2–3 generations per year in contrast to the high-altitude populations which are univoltine (Tolman and Lewington 2008). Thus, the time constraints imposed by having to fit in additional generations a year seem to be much more severe than those imposed by the shorter vegetation period in alpine environments (Roff 1980; Nylin and Gotthard 1998; Burke et al. 2005; Karl et al. 2008). Interestingly, Estonian populations of *L. tityrus* also show shorter development times (and smaller body size) compared to German populations, presumably as an adaptation to the short season length in Estonia (Reim et al. 2018b). However, Estonian populations have two generations a year, supporting the above conclusions (Reim et al. 2018b). Interestingly, the differences in larval development time were restricted to the lower rearing temperature, at which also the difference in larval growth rate was more pronounced. This may indicate that the low-altitude populations speed up their development as much as possible if ambient conditions are challenging for quick development (Nylin and Gotthard 1998), a pressure univoltine high-altitude populations do not face.

Further evidence for local adaptation stems from the fact that high-altitude populations gained a higher body mass than the low-altitude ones at the lower rearing temperature, but vice versa at the higher rearing temperature. Thus, animals were most efficient under the thermal conditions they are presumably better adapted to, probably reflecting metabolic cold adaptation and associated costs at warmer temperatures in high-altitude populations (see Addo-Bediako et al. 2002; Terblanche et al. 2009).

High-altitude populations seem to invest less into dispersal, having smaller wings (despite a similar body mass) and concomitantly higher wing loadings, lower wing aspect ratios and relative abdomen fat contents, and tending to have lower thorax masses as compared with low-altitude populations. Typically, large thoraxes and wings as well as low wing loadings and aspect ratios indicate a high dispersal ability and an aerodynamically- and cost-efficient flight (Berwaerts et al. 2002; Almbro and Kullberg 2012; Lion et al. 2023), while abdominal fat is used to fuel dispersal (Arrese and Soulages 2010; Toprak et al. 2020). However, in general insects seem to have increased rather than decreased wing sizes at higher altitudes (Dillon et al. 2006). The opposite pattern revealed in our study, i.e., smaller wings and a lower investment into dispersal in high-altitude populations, might result from inhabiting relatively continuous habitats along alpine streams (Trense et al. 2021), and/or from a lower dispersal propensity due to an exposure to stronger winds at higher altitudes (Hodkinson 2005).

In general, insect herbivores are often nitrogen limited, typically referred to as the nitrogen limitation hypothesis (White 1993). However, in our study, high levels of host plant fertilization as used in modern agriculture, thus exceeding natural nutrient levels, negatively affected fitness components in *L. tityrus*, reducing growth rates, body mass and concomitantly wing loading, and prolonging larval time (cf. Fischer and Fiedler 2000; Kurze et al. 2018; Raharivololoniaina et al. 2023). Note that low levels of host plant nitrogen fertilization often increases insect fitness in line with nitrogen limitation hypothesis (White 1993; Kurze et al. 2017; Raharivololoniaina et al. 2021), while high levels may exert negative effects. Thus, herbivore performance in response to host plant nitrogen levels likely follows a hump-shaped curve (Han et al. 2014; Tao et al. 2014; Lebigre et al. 2018). Such negative effects may even include increased mortality (Fischer and Fiedler 2000), though here we could only find sublethal effects.

Consequently, *L. tityrus* seems highly vulnerable to agricultural intensification not only due to more frequent grassland cutting or more intensive grazing, but also due to increasing nutrient loads. Possible mechanisms behind negative effects of increased nitrogen fertilization on insect herbivores include host-plant changes in elemental stoichiometry, nutritional geometry, essential micronutrients, and allelochemicals (Vogels et al. 2023). As predicted, detrimental effects were more pronounced in high-altitude populations, such that these populations, likely adapted to low levels of host-plant nitrogen, are even more vulnerable to agricultural intensification. Note that all alpine populations were sampled in the central Alps on unfertilized meadows above crystalline primary rock, resulting in acidic, nutrient-poor soils. In contrast, sampling locations of low-altitude populations were on partly fertilized meadows above less acidic soils (basalt, marshland). This also suggests that our data probably do not reflect recent adaptations to different levels of nitrogen availability, but rather long-term local adaptation. In any case, our results are in agreement with the notion that herbivores from naturally nutrient-poor environments should be more strongly affected than those from richer environments (Vogels et al. 2023). Furthermore, detrimental effects of nitrogen fertilization were more pronounced at the higher rearing temperature, indicating increasing risks from climate change. Similarly, additive effects between host-plant nitrogen fertilization and drought were found in *L. tityrus* (Raharivololoniaina et al. 2023).

The effects of sex and temperature on life-history traits were as expected. Female insects are typically selected for large body size and a high investment into the abdomen, which is positively related to fecundity (Honek 1993; Nylin and Gotthard 1998; Teder et al. 2021). Males, in contrast, have a selective premium on rapid development to eclose before the females (protandry) and on flight capacity, both increasing their reproductive success (Wiklund et al. 1991; Berwaerts et al. 2006; Reim et al. 2018b). As generally found in ectotherms, development time was longer and growth rates lower at the lower developmental temperature (Von Bertalanffy 1960; Karl and Fischer 2008). According to the temperature-size rule on plasticity in body size (Atkinson and Sibly 1997; Karl and Fischer 2008), animals were larger at the lower temperature, also resulting in higher wing loadings. The higher mortality rates at the lower rearing temperature are likely associated with the concomitantly much longer development time.

In summary, we show local adaptation in developmental traits in the butterfly *L. tityrus*. Low-altitude populations were adapted to warmer temperatures and longer seasons, displaying a more rapid development and phenotypes indicative of a high dispersal capacity. Importantly, we also show that *L. tityrus* is vulnerable to agricultural intensification, responding negatively to agriculturally relevant levels of nitrogen fertilization of its host plant. According effects were particularly pronounced at warmer temperatures and in high-altitude populations. Thus, our study adds to the increasing knowledge that different drivers of global change, here nutrient loading and climate change, may interact and thereby increase the overall level of threat to biodiversity (Raven and Wagner 2021). Furthermore, our study suggests that populations inhabiting nitrogen-poor environments, as can be assumed for the alpine populations investigated here, might be even more vulnerable to agricultural intensification than others, which may be applicable to many species (see also Turlure et al. 2013). A much lower level of nitrogen input in the habitats of the alpine populations investigated here compared to those of the low-altitude populations is very likely, resulting from an absence of any fertilization of these remote alpine meadows in combination with acidic ground rocks and a low level of atmospheric nitrogen deposition in contrast to central and northern Germany (Walter 2007; European Environment Agency 2024).

In *L. tityrus*, high-altitude populations seem to be especially vulnerable, showing a high sensitivity to high nitrogen levels, reduced dispersal capacity, and reduced plasticity in heat resistance (Karl et al. 2008, 2009b). We think that our findings, specifically the detrimental effects of agriculturally relevant levels of nitrogen fertilization being exaggerated at warmer temperatures and in populations from nutrient-poorer environments, may have important implications for other vulnerable species in the face of rapid environmental change. In particular, nitrogen fertilization and deposition should be reduced especially in naturally nutrient-poor environments. Future research should investigate whether according species, populations, and communities are indeed more vulnerable to nutrient input, and which mechanisms may be responsible for detrimental effects.

## Supplementary Information

Below is the link to the electronic supplementary material.

Supplementary file1 (DOCX 29 KB)

## Data Availability

The datasets used during the current study are available from the corresponding author on reasonable request.
