# Supplementary material for: Negative effects of nitrogen fertilization on herbivore fitness are exaggerated at warmer temperatures and in high-altitude populations
Source: Oecologia. 2025 Mar 11;207(3):51. doi: 10.1007/s00442-025-05690-8 (PMC11893656; doi:10.1007/s00442-025-05690-8)
Supplement: Supplementary file 1 — Supplementary file1 (DOCX 29 KB) [file 442_2025_5690_MOESM1_ESM.docx]

**Supplementary material**

**Table S1** Effects of temperature and sex (F: female, M: male) on abdomen mass, wing loading, wing length and wing aspect ratio (a), and effects of origin (high vs. low altitude) and sex on thorax mass, wing length, and wing loading (b) in the butterfly *Lycaena tityrus.* Given are means ± SE.

| **(a)** |  |  |  |  |  |
| --- | --- | --- | --- | --- | --- |
| **Temp.** | **Sex** | **Abdomen mass**  [mg] | **Wing loading**  [mg/mm^2^] | **Wing length**  [mm] | **Wing aspect** |
| 19°C | F | 18.07 ± 0.42 | 0.447 ± 0.009 | 15.07 ± 0.10 | 9.37 ± 0.09 |
| 19°C | M | 9.13 ± 0.42 | 0.358 ± 0.009 | 14.95 ± 0.10 | 10.16 ± 0.09 |
| 25°C | F | 13.12 ± 0.26 | 0.344 ± 0.005 | 14.84 ± 0.06 | 9.28 ± 0.05 |
| 25°C | M | 6.74 ± 0.23 | 0.289 ± 0.005 | 14.55 ± 0.05 | 9.80 ± 0.05 |
| **(b)** |  |  |  |  |  |
| **Origin** | **Sex** | **Thorax mass**  [mg] | **Wing length**  [mm] | **Wing loading**  [mg/mm] |  |
| High | F | 15.38 ± 0.41 | 14.47 ± 0.09 | 0.412 ± 0.008 |  |
| High | M | 13.95 ± 0.42 | 14.42 ± 0.09 | 0.322 ± 0.008 |  |
| Low | F | 15.67 ± 0.36 | 15.44 ± 0.07 | 0.375 ± 0.007 |  |
| Low | M | 15.38 ± 0.31 | 15.09 ± 0.07 | 0.325 ± 0.006 |  |

**Table S2** Effects of origin (low versus high altitude), temperature, and host-plant nitrogen treatment on wing length, wing area and pupal mass in the butterfly *Lycaena tityrus*. Given are means ± SE.

| **Origin** | **Temp.** | **Nitrogen** | **Wing length**  [mm] | **Wing area**  [mm^2^] | **Pupal mass**  [mg] |
| --- | --- | --- | --- | --- | --- |
| High | 19°C | Low | 14.44 ± 0.15 | 85.25 ± 1.76 | 116.96 ± 3.31 |
| High | 19°C | High | 14.83 ± 0.17 | 93.04 ± 2.00 | 126.49 ± 3.66 |
| High | 25°C | Low | 14.39 ± 0.07 | 88.09 ± 0.87 | 116.58 ± 1.62 |
| High | 25°C | High | 14.12 ± 0.09 | 85.43 ± 1.05 | 114.72 ± 1.97 |
| Low | 19°C | Low | 15.41 ± 0.10 | 96.77 ± 1.25 | 124.94 ± 2.35 |
| Low | 19°C | High | 15.37 ± 0.13 | 98.12 ± 1.52 | 126.17 ± 2.85 |
| Low | 25°C | Low | 15.18 ± 0.07 | 95.41 ± 0.85 | 125.57 ± 1.60 |
| Low | 25°C | High | 15.09 ± 0.09 | 95.52 ± 1.04 | 127.04 ± 1.95 |
